# Instrumental variable approach on analyzing risk factors associated with noncommunicable disease prevalence in Tanzania: A nonexperimental design

**DOI:** 10.1002/hsr2.1585

**Published:** 2023-09-28

**Authors:** Felician A. Kitole, Jennifer K. Sesabo, Robert M. Lihawa

**Affiliations:** ^1^ Department of Economics Mzumbe University Morogoro Tanzania

**Keywords:** control function approach, disease burden, instrumental variable, noncommunicable diseases (NCDs), public health, two stage residual inclusion

## Abstract

**Background and Aims:**

Noncommunicable diseases (NCDs) have emerged as a substantial burden in developing countries, representing the leading cause of mortality. Addressing this critical issue necessitates effective interventions and policy measures. Therefore, this study aims to investigate the risk factors associated with NCD prevalence in Tanzania.

**Methods:**

This study employed a nonexperimental research design due to its ability to analyze secondary data without altering variables. The used data set of the study was sourced from National Panel Survey 2020/21 and Household Budget Survey 2017/18. The econometrics analysis applied in the study include two‐stage residual inclusion (2SRI) and control function approach due to their ability to suppress endogeneity and enhance the clarity of results.

**Results:**

The findings indicate a significant positive correlation between alcohol consumption (0.4110382, *p* = 0.02), cigarette smoking (0.3354297, *p* < 0.001), and NCDs, emphasizing the urgency of targeted interventions to mitigate these behaviors. Conversely, a negative correlation is observed between fruit and vegetable intake (−0.1063375, *p* < 0.001), physical exercises (−0.3744925, *p* < 0.001), and NCDs, underscoring the importance of promoting healthy dietary habits and frequent exercises.

**Conclusion:**

These results accentuate the immediate need for targeted interventions and policy measures to address these risk factors and effectively combat the escalating burden of NCDs in Tanzania and similar contexts. Moreover, the need for improved public awareness campaigns and the promotion of healthy life campaigns are vital in the fight to lower the prevalence of NCDs across communities.

## Key points

- While the global share of noncommunicable diseases (NCDs) deaths is uncontrollable, the disease accounts for 34% of total deaths in Tanzania.
- Adequate physical exercises, fruit and vegetable intake protects against NCDs, while alcohol, cigarette smoking, and NCDs have strong positive correlations.
- Urban residence, age, and sex vary in their correlation with NCD risk, highlighting the complexity of NCD determinants.
- Poor healthcare infrastructure makes NCDs more vulnerable, emphasizing the need to improve it.
- Public health campaigns, health education, healthcare system strengthening, and evidence‐based policies are needed to reduce NCD prevalence, promote healthier lifestyles, and reduce risk factors.

## 1 INTRODUCTION

In a world with limited resources, prioritizing becomes a crucial endeavor, extending to the field of public health.^1^ Together with an alarming prevalence of noncommunicable diseases (NCDs), the contemporary global landscape is witnessing an unprecedented rise in the elderly population. This context highlights the significance of risk reduction, NCD prevention, and healthy ageing promotion strategies. Over the past few decades, there has been a substantial expansion in the study of dementia risk factors. While early research focused on individual risk factors using clinical trials and evidence synthesis, there has recently been a paradigm shift toward understanding the collective impact of multiple risk factors and developing multifactorial risk reduction strategies.^1^, ^2^, ^3^

NCDs pose a formidable threat to global health, accounting for 74% of global deaths, with 86% of premature deaths occurring in low‐ and middle‐income countries.^4^, ^5^ The trajectory of African public health initiatives has primarily centered on communicable diseases. However, an alarming increase in premature mortality and disability due to NCDs and mental health conditions requires a shift in focus. The proportion of total DALYs attributable to NCDs in Sub‐Saharan Africa increased from 18% to 30% between 1990 and 2017.^6^ This transition to a “triple burden” scenario, which includes communicable diseases, NCDs, and injuries, poses a difficult challenge.

Tanzania is not immune to the looming shadow of lifestyle‐related NCDs. The 2022 Noncommunicable Disease Progress Monitor report revealed that NCDs account for 34% of annual Tanzanian deaths or an average of 110,600 deaths annually. Concerning 17% of premature mortality risk is attributed to NCDs.^4^, ^6^ Efforts by the Tanzanian government and global organizations, exemplified by the World Health Organization, have yielded numerous strategies aimed at public awareness, improved healthcare infrastructure, and targeted policies to address lifestyle factors that contribute to NCDs.^7^, ^8^

Despite these commendable efforts, the attainment of NCD mitigation objectives remains incomplete, primarily due to a lack of emphasis on NCD mitigation on a national scale. This gap is fueled by a limited understanding and empirical limitations in identifying immediate risk factors and holistic repercussions of NCDs ranging from individual households to the national economy. It emphasizes the need for extensive, rigorous scientific research into the intricate complexities of NCDs. Mayige et al.,^9^ Andrew,^8^ Kitole et al.,^10^ and Kitole et al.^7^ have laid the groundwork; however, additional research is required to decipher the complex web of variables influencing NCD prevalence and its far‐reaching consequences.

The diverse array of risk factors associated with NCDs has been illuminated by empirical research. Andrew^8^ and Kitole et al.^4^ highlight the prevalence of unhealthy lifestyles that contribute to the escalation of NCDs. Significantly, social interactions foster the imitation of behaviors that influence the emergence and prevalence of NCDs. Unwin et al.^11^ note that unregulated habits, such as excessive or insufficient consumption, are significant contributors to reported cases of NCD. According to the World Health Organization, NCDs will account for approximately 77% of deaths between 1990 and 2020 due to urbanization and lifestyle changes.

In addition, studies highlight dietary habits, smoking, alcohol consumption, and insufficient physical activity as key risk factors for NCDs.^12^ Overconsumption of salt, calories, and saturated fat exacerbates the burden of NCDs.^13^ The correlation between excessive salt consumption and hypertension, which results in 59% of deaths in low‐income countries, demonstrates the gravity of the problem.^14^ Cholesterol levels are exacerbated by insufficient physical activity, which increases the likelihood of NCDs.^15^, ^16^

The impact of NCD risk factors differs by country and cluster. There is a correlation between poverty and underweight births, and chronic NCDs.^17^, ^18^, ^19^ Sex, age, marital status, household size, and ethnicity are dynamic contributors to NCD prevalence.^20^, ^21^ Hypertension exemplifies hereditary characteristics contributing to familial susceptibility.^22^, ^23^ Diverse factors, including physical activity, diet, toxins, and urban stress, can explain urban‐rural disparities in NCD prevalence.^24^, ^25^ Nevertheless, comprehensive analyses frequently overlook social interactions and hereditary factors, highlighting the importance of the present study's investigation.

The present study incorporates unobserved effects of social interactions and hereditary components to examine their implications on NCD risk factors across individuals' locality and family backgrounds. Thus, the study introduces social membership engagement/participation as a proxy for social interactions, shedding light on the complex dynamics that influence health outcomes.

## 2 METHODS AND DATA

This study used a nonexperimental research design because of its inherent capacity to utilize secondary data from the Tanzania National Bureau of Statistics. The data sets consist of the National Panel Survey Wave 5 of 2020/21 and the Household Budget Survey 2017/18, combined to enhance clarity and incorporate key insights for elucidating the prevalence of NCDs across diverse communities in Tanzania. These data sets contain abundant socioeconomic and health‐related information pertinent to the study's objectives (see Table 1).

**Table 1:** Definition and measurement of variables.

Variable	Definition	Measurement
Age	Number of years a person lived	Continuous variable
Age squared	Age squared	Continuous variable
		“Age squared”
Residence	Location of household residence	Dummy
		“Urban = 1 and 0 = otherwise”
Sex	Biological characteristics of a person	Dummy
		“Male = 1 and 0 = otherwise”
Household income	Total household income	Continuous variable
		“Log total household income”
Years of schooling	Number of years a person spent on schooling	Continuous variable
		“Total number of years in schooling”
Alcohol	Regularity of alcohol usage status	Dummy
		“Drink alcohol regularly = 1 and 0 = otherwise”
Cigarette	Regularity of cigarette smoking status	Dummy
		“Smoke cigarette regularly = 1 and 0 = otherwise”
Fruits and vegetables	Regularity of fruit and vegetable consumption status	Dummy
		“Consume fruits and vegetables regularly = 1 and 0 = otherwise”
Alcohol consumption	The average amount of alcohol consumed	Continuous variable
Vegetable and fruits consumption	The average amount of vegetables and fruits consumed	Continuous variable
Cigarette consumption	The average amount of cigarettes consumed	Continuous variable
Distance to the nearest health facility	Distance to the nearest health facility in km	Continuous variable
Physical exercises	Number of times a person engages in physical activities in a day	Continuous variable
Level of education	The highest level of education attained	Not attended school
		Primary education
		Secondary education
		College education
		Higher learning education
Hereditary disease	Presence of hereditary diseases	Dummy
		“Yes = 1 and 0 = otherwise”
Social membership	Member of any social group	Dummy
		“Member = 1 and 0 = otherwise”
Noncommunicable diseases (NCD)	Presence of noncommunicable disease victims in a household	Dummy
		“Urban = 1 and 0 = otherwise”

**Figure 1:**
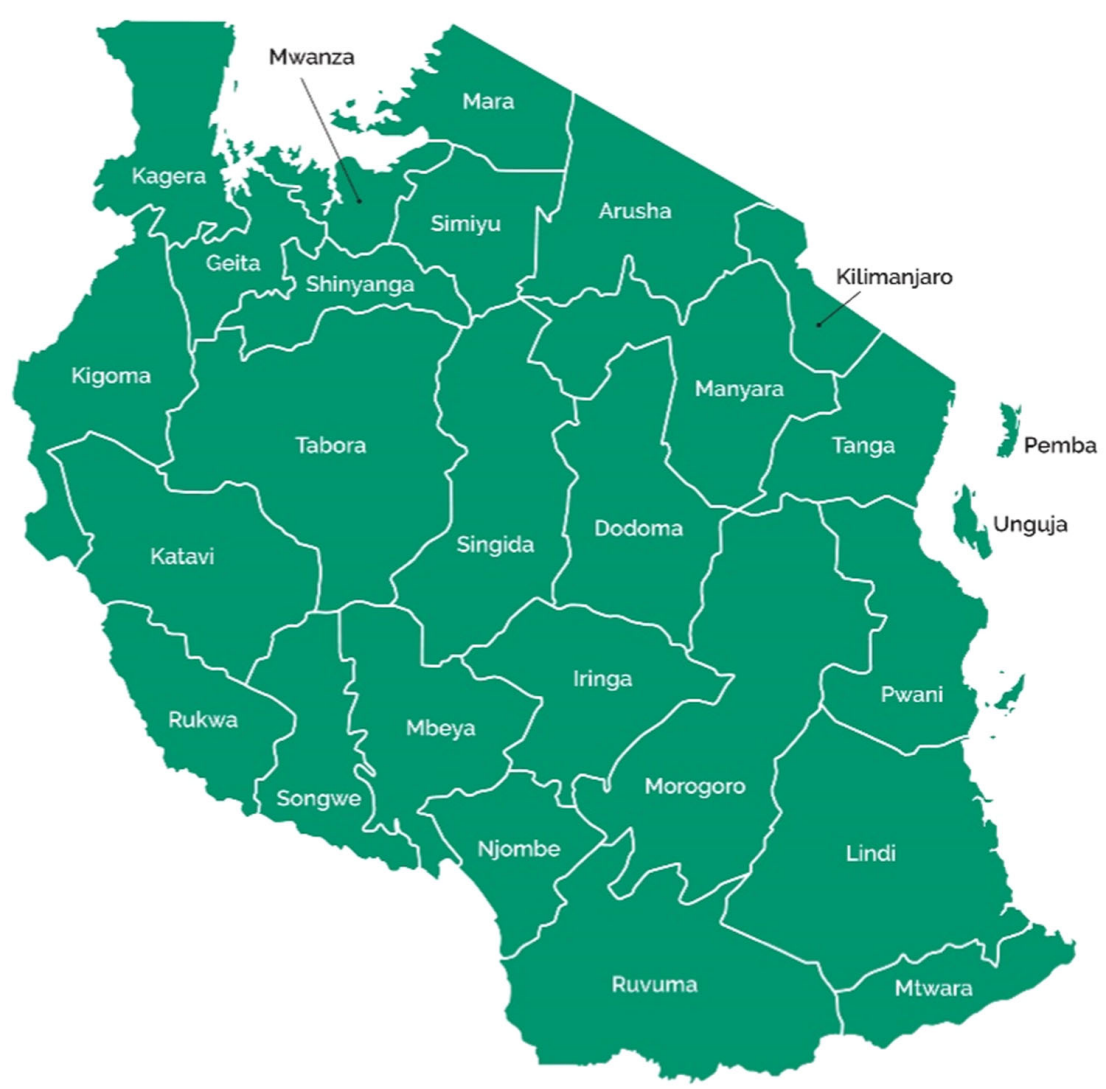
The map of Tanzania showing regions.

In contrast, previous research efforts, such as those by Andrew,^8^ Kitole et al.^4^ in Tanzania, and Mwai and Muriithi^14^ in Kenya, were limited by their reliance on single survey data sets, thereby limiting the examination of essential household‐level characteristics. Notably, the study focused on the binary measurement of NCD status. In this case, the dummy variable was assigned to households that reported any major NCDs within the previous 12 months. Figure 1 provides additional geographical context by depicting a map of Tanzania's study area.

In tandem, the study utilized the analytical prowess of STATA 17.0 for coding, data management, and rigorous statistical analysis. The approach to analysis included both descriptive and inferential statistics. Through tabulated frequencies, means, and standard deviations, descriptive statistics skillfully portrayed the fundamental attributes and characteristics of the data set. Collectively, these analytic foundations bolster the exhaustive examination of the research questions and provide a solid and methodologically sound framework for the investigation.

In contrast, the instrumental variable models of two‐stage residual inclusion (2SRI) and control function approach (CFA) were used to analyze the risk factors for NCDs, where risks factors, particularly income, were regarded as an endogenous variable as it explains other risks factors like cigarette smoking and alcohol consumption, therefore to suppress endogeneity, the distance to the nearest water sources was used as an instrument. The used models (2SRI and CFA) assume that the relationship between the endogenous variable and the dependent variable is nonlinear and that a confounding variable (distance to a water source) affects the endogenous variable but not the dependent variable directly.^5^, ^26^, ^27^

The general structural equation that explains the relationship can be written as;

(1) $$\text{Pr}\left(Y=1│X,Z\right)=\varphi \left(\beta_{0}+\beta_{1}X+\beta_{2}Z+\varepsilon \right)$$

Where Y is the NCD status, X is the endogenous variable (income), Z is the instrumental variable (distance to the nearest water source), and $\beta_{0}$, $\beta_{1}$, and $\beta_{2}$ are the model parameters, ε is the error term, φ is the cumulative distribution function of the standard normal distribution.

The relationship between the instrumental variable (Z) and the endogenous variable is estimated using the first‐stage regression. The estimated coefficient of the instrumental variable ($\beta_{2}$) is used to calculate the expected value of the endogenous variable ($\hat{X}$), which is then included as an independent variable in the second stage of regression with the dependent variable (Y) and other control variables (X).

The first stage regression is expressed as;

(2) $$X=Y_{0}+Y_{1}Z+\delta$$

Whereas $Y_{0}$ and $Y_{1}$ are the first‐stage regression parameters, and δ is the error term. The predicted values of the endogenous variable ($\hat{X}$) is obtained from;

(3) $$\hat{X}=Y_{0}+Y_{1}Z$$

The second stage regression is then rewritten as;

(4) $$\text{Pr}\left(Y=1│\hat{X},X,W\right)=\varphi \left(\alpha_{0}+\alpha_{1}\hat{X}+\alpha_{2}X+\alpha_{3}W+\mu \right)$$

Whereas W represent a vector of control variables, $\alpha_{0},\alpha_{1},\alpha_{2}$, and $\alpha_{3}$ are model parameters, and μ is the error term.

The results of the instrumental variable models provide estimates of the influence of the endogenous variable on the dependent variable while controlling for the confounding variable (distance to a water source) and other control factors (W). The validity of the instrumental variable model is based on the premise that the instrumental variable (distance to a water source) is significant and exogenous, meaning that it influences the endogenous variable but not the dependent variable. In addition, the model implies that no unobserved confounding variables influence both the endogenous and dependent variables.

Furthermore, the presence of endogeneity concerns is determined not by the researchers' subjective opinions, but rather by rigorous statistical tests that provide justification for employing instrumental variable models. As shown in Appendix 1, the null hypothesis of exogeneity was rejected in favor of endogeneity (p=0.005) emphasizing that there is endogeneity in the model. In testing the instrument's validity, which is the distance to the water source, the instrument was chosen based on its ability to satisfy key conditions: it has causal effects on exogenous variables, affects the outcome only through exogenous variables, and lacks confounding effects. Appendix 2 presents summary statistics for the first‐stage regression of the distance to the water source. The *R* ^2^ value of 0.6537 (indicates that this variable explains about 65.37% of exogenous variables' variation, with an adjusted *R* ^2^ value of 0.5621). The partial *R* ^2^ value of 0.5388 suggests a significant relationship between distance to the water source and exogenous variables, supported by the *p*‐value of 0.0000. These findings affirm the instrument's strength.

The limited information maximum likelihood method was employed, with Appendix 3 showing the instrument's evaluation. The rejection rate for the Wald test does not exceed 10% (5% nominal level), and the eigenvalue statistic of 57.89 surpasses critical values even at 30%, indicating no weak instrument problem. The instrument solely exerts indirect effects through the treatment variable. The Sargan test and Basmann test in Appendix 4 exhibit high *p*‐values with the test statistics of 35.0042 (p>0.99) and, 28.6433 (p>0.99), respectively, suggesting no evidence to reject the null hypothesis of invalid instruments. Thus, these tests support the validity of the instruments used in the analysis.

## 3 FINDINGS

Findings in Table 2 show that 8551 out of 25,730 people, equivalent to 33%, are suffering from NCDs, while only 67% are not suffering from these diseases. Moreover, results stipulate that 2708 (32%) out of 8551 victims of NCDs are hereditary, while 5843 (68%) are the result of nonhereditary factors, which include lifestyles. The prevalence of NCDs based on locality has shown that only 3264 (38%) of the victims live in rural areas while the majority (62%) live in urban areas. Additionally, findings in Table 2 show that most of the NCD victims are females (60%) while the affected males are just 40% of entire NCDs. Furthermore, findings in Table 2 show that non‐members in social groups are highly affected by NCDs compared to members. This justifies that social groups can help members get information on diseases and reduce the chances for members to be affected.

**Table 2:** NCDs across demographic and biological factors.

Variables	Attributes	NCD victims	Non‐victims	Total	*χ* ^2^	p
Hereditary	Yes	2708 (32%)	3149 (18%)	5857	11.5623	0.014
	No	5843 (68%)	14,030 (82%)	19,873		
	Total	8551 (100%)	17,179 (100%)	25,730		
Residence	Rural	3264 (38%)	12,406 (72%)	15,670		<0.001
	Urban	5287 (62%)	4773 (285)	10,060		
	Total	8551 (100%)	17,179 (100%)	25,730		
Sex	Male	3394 (40%)	8956 (52%)	12,350	35.21	<0.001
	Female	5157 (60%)	8223 (48%)	13,380		
	Total	8551 (100%)	17,179 (100%)	25,730		
Social membership	Members	1052 (12%)	7933 (46%)	8985	19.023	<0.001
	Non‐members	7499 (88%)	9246 (54%)	16,745		
	Total	8551 (100%)	17,179 (100%)	25,730		

Abbreviation: NCDs, noncommunicable diseases.

**Figure 2:**
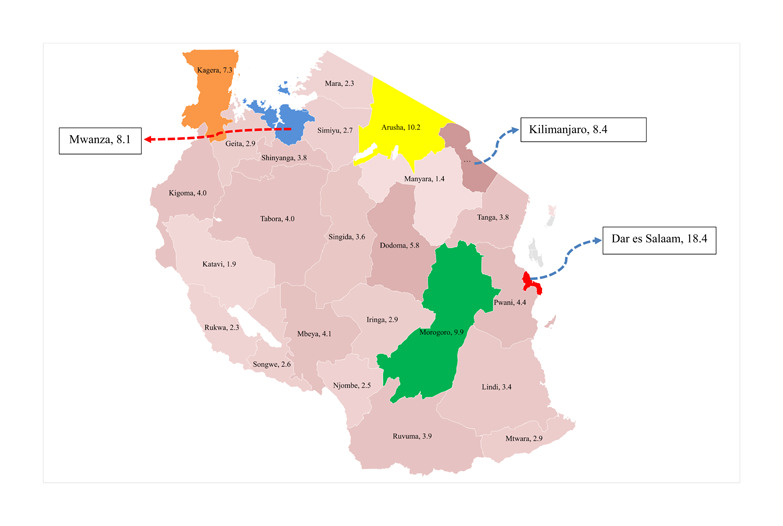
Prevalence of noncommunicable diseases (NCDs) among regions in Tanzania, (*n* = 11,707). *Source*: Author's computation, 2023.

The study's findings in Figure 2 show that the Dar es Salaam region (red‐shaded) had the highest prevalence of NCDs in Tanzania at 18.4%. Dar es Salaam is a famous trading city in Africa which is highly urbanized in the East African region and contributes more than 70% of the entire GDP in Tanzania. Therefore, the higher prevalence of NCDs justifies that NCDs hit more urban residents; this is why NCDs have been termed as urban diseases.

On the other hand, other regions with a higher prevalence of NCDs are Arusha (10.2%), Morogoro (9.9%), Kilimanjaro (8.4%), Mwanza (8.1%), and Kagera (7.3%). Moreover, the southern zone of the country had the lowest prevalence of which Lindi had 3.4%, Mtwara (2.9%), and Ruvuma has 3.9%; findings on the NCDs prevalence for other regions are shown in Figure 2.

Moreover, findings in Table 3 show that the proportion of female‐headed households was 52% while that of males was 48%, of which 60.9% of entire households resides in rural areas. In comparison, the remaining 39.1% reside in urban areas. In addition, only 29.4% of entire households consume vegetables and fruits regularly, while the majority 70.6% do not consume vegetables and fruits regularly. It indicates that most households do not tend to consume vegetables and fruits during their meals. Vegetables and fruits are rich in vitamins and important minerals for body repairs, growth, and development.

**Table 3:** Social demographic characteristics on various parameters (*n* = 25,750).

Characteristics	Measurement	Frequency	Percentage (%)
Sex	Female	13,380	52
	Male	12,350	48
Place of residence	Urban	10,060	39.1
	Rural	15,670	60.9
Vegetable and fruits consumption	Regular consumers of vegetables and fruits	7565	29.4
	Doesn't consume vegetables and fruits regularly	18,165	70.6
Alcohol consumption	Consumes alcohol	11,810	45.9
	Does not consume alcohol	13,920	54.1
Cigarette smoking	Smokes cigarettes	12,273	47.7
	Does not smoke cigarettes	13,457	52.3
NCD	Has an NCD	11,707	45.5
	Does not have any NCD	14,023	54.5
Marital status	Married	15,850	61.6
	Single	9880	38.4
Level of education	Not attended school	1660	6.45
	Primary	13,097	50.90
	Secondary	7204	28.00
	College	2766	10.75
	University	1003	3.90
Age	Maximum	84	
	Mean	42.41	
	Standard deviation	12.29	
Household income	Maximum	133,000,000	
	Mean	896,378	
	Standard deviation	1,652,771	
Physical exercises	Maximum	10	
	Mean	0.60573	
	Standard deviation	0.05856	

Abbreviation: NCDs, noncommunicable diseases.

*Source*: Author's computation, 2023.

On the other hand, the proportion of heads of households that consume alcohol was 45.9%, while those who do not consume were just 54.1%, while the proportion of cigarette smokers was 47.7%. These findings indicate that most heads of households in Tanzania are engaging in the consumption of commodities that endanger their health toward NCDs.

In addition, findings show that most heads of households (50.9%) have attended primary education as their highest level; those with secondary education were 28.00%, college was 10.75%, the university was 3.90%. In contrast, those with no educational background (not schooled) were 6.45% of the population under the study. Education information shows household knowledge and awareness toward diseases, especially NCDs. Kitole et al.^5^ argue that the fact that NCDs develop slowly and emerge to be critical with time when people have no sufficient educational background and the ability to detect early symptoms increases the disease's prevalence. In cementing these arguments, Oyebode et al.^28^ added that low education and lack of health knowledge have led to NCD cases in most rural areas in developing countries, making NCDs no longer urban diseases.

**Figure 3:**
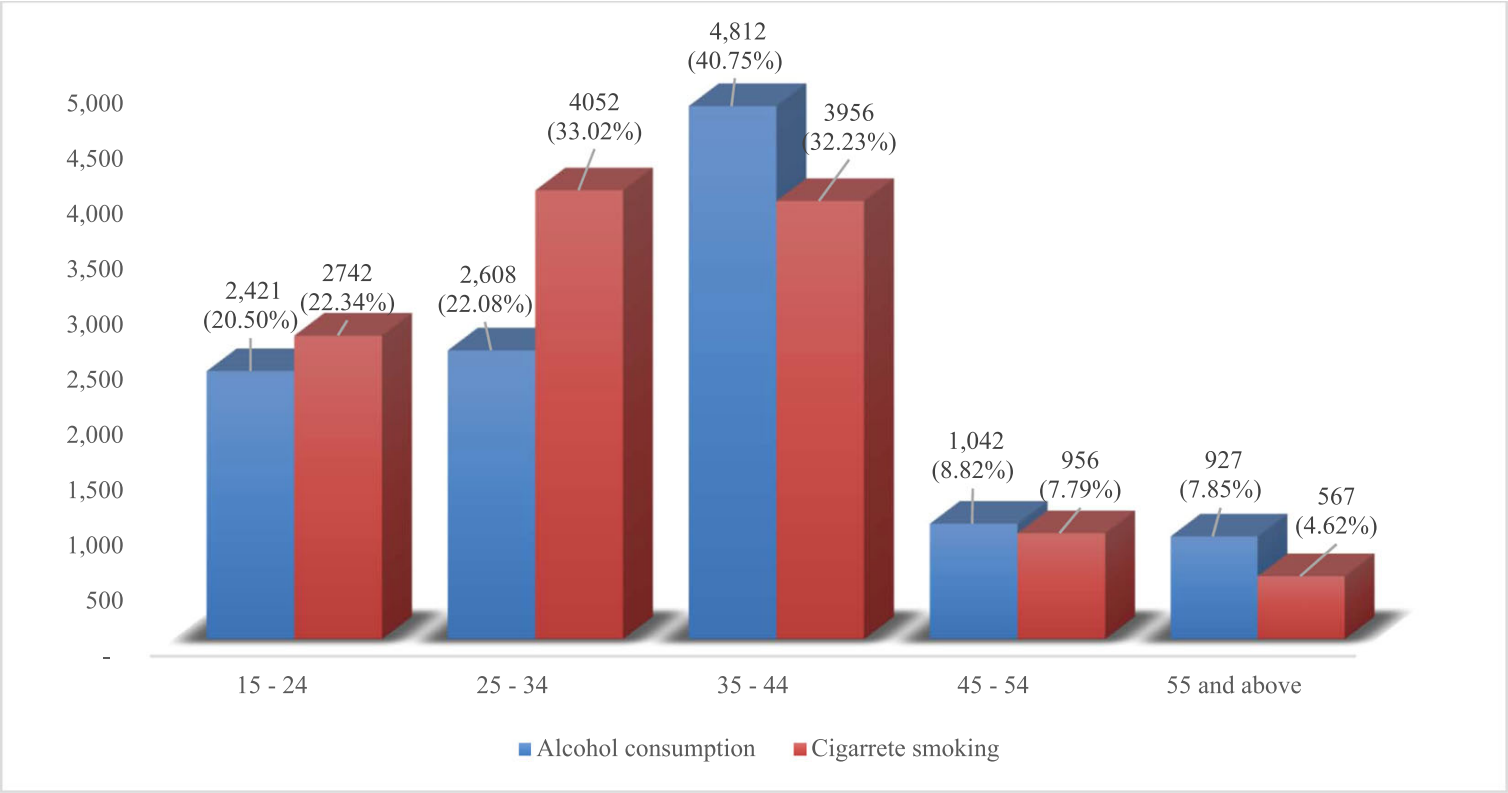
The alcohol consumption (*n* = 11,810) and cigarette smoking (*n* = 12,273) across age cohorts.

Figure 3 depicts the distribution of alcohol consumption and cigarette smoking across various age cohorts. Within the demographic of individuals between the ages of 15 and 24, approximately 20.50% acknowledged engaging in alcohol consumption, whereas 22.34% admitted to cigarette smoking. Among individuals aged 25−34, alcohol consumption was slightly elevated, accounting for 22.08% of the population. Conversely, the prevalence of cigarette smoking was comparatively higher, reaching 33.02%. Among individuals aged 35−44, the prevalence of alcohol consumption exhibited an upward trend, reaching 40.75%.

Additionally, 32.23% of individuals in this age group reported smoking cigarettes. A diminished occurrence of alcohol consumption was noted among individuals aged 45−54, with a prevalence rate of 8.82%. Additionally, 7.79% of individuals in this age group reported smoking cigarettes. Within the cohort of individuals aged 55 and older, a notable proportion of 7.85% disclosed engaging in alcohol consumption, while a comparatively smaller subset of 4.62% acknowledged their involvement in cigarette smoking.

The findings in Table 4 present the results obtained from estimating Equations 1 and 2, which sheds light on the influence of presumed risk factors on NCDs. At the same time, Columns 2 and 3 demonstrate the estimates utilizing the 2SRI and CF approaches, respectively, to account for endogeneity and heterogeneity. The findings presented in Table 4 were used to examine heterogeneity and endogeneity by assessing residuals using the 2SRI regression. The results validate the existence of endogeneity in the equation, as demonstrated by the significant residuals associated with fruits and vegetables, alcohol consumption, and cigarette smoking. These significant residuals indicate the influence of unobservable factors that contribute to the occurrence of NCDs, factors which remain unknown to the researcher.

**Table 4:** Contribution of specific risk factors to NCDs prevalence in Tanzania.

Explanatory variables	Estimation methods					
	Probit (1)	*p* Value	2SRI (2)	*p* Value	CFA (3)	*p* Value
Age	0.0434399 (0.00068)	p<0.001	0.1288561 (0.000023)	p<0.001	0.1511301 (0.0000172)	p<0.001
Age squared	0.0102184 (0.00016)	p<0.001	−0.0418160 (0.0025034)	p=0.02	−0.007313 (0.0008002)	p=0.04
Urban	0.0664567 (0.010524)	p=0.05	0.1044684 (0.00848707)	p=0.03	0.15652009 (0.0083328)	p<0.001
Sex (male)	−0.0786953 (0.15652)	p>0.99	−0.1367008 (0.1825644)	p>0.99	−0.3338514 (0.2091832)	p>0.99
Household income	0.1465603 (0.04831)	p<0.001	0.1574776 (0.1646561)	p<0.001	0.2035366 (0.0013272)	p<0.001
Years of schooling	0.0033381 (0.41654)	p>0.99	0.0092593 (0.361063)	p>0.99	0.0426109 (0.2617970)	p>0.99
Alcohol dummy	0.1672995 (0.158606)	p>0.99	0.3350415 (0.1137595)	p=0.04	0.4110382 (0.1089123)	p=0.02
Cigarette dummy	0.2649082 (0.100436)	p=0.03	0.2726841 (0.0657126)	p=0.02	0.3354297 (0.0822663)	p<0.001
Fruits/vegetable dummy	−0.0219370 (0.029500)	p>0.99	−0.0628739 (0.0160226)	p=0.04	−0.1063375 (0.0073299)	p<0.001
Average alcohol consumption	0.0027892 (0.000781)	p=0.01	0.0073497 (0.0005052)	p=0.03	0.0086479 (0.0037229)	p=0.02
Average vegetables and fruits consumption	−0.0030276 (0.00093)	p=0.002	−0.0070918 (0.0031315)	p=0.006	−0.0083577 (0.0034191)	p=0.004
Average cigarette consumption	0.0059421 (0.00141)	p<0.001	0.0146029 (0.0048019)	p<0.001	0.018882 (0.0065293)	p<0.001
Distance to the health facility	0.0208342 (0.01125)	p=0.05	0.0974814 (0.0135012)	p=0.03	0.1178129 (0.001632)	p=0.02
Hereditary	0.0399275 (0.000204)	p=0.04	0.0960433 (0.00270)	p=0.03	0.199654 (0.00165)	p<0.001
Physical exercise	−0.2968853 (0.08559)	p=0.02	−0.3241512 (0.116191)	p=0.01	−0.3744925 (0.010977)	p<0.001
Alcohol residual			−0.1274317 (0.2512781)	p>0.99	−0.606732 (0.4730524)	p>0.99
Cigarette residual			−0.6388615 (0.2722084)	p=0.03	−0.5079913 (0.2199268)	p=0.01
Fruits and vegetables' residual			0.7212467 (0.2748135)	p=0.04	0.7002382 (0.3008106)	p=0.02
Fruits and vegetables × residual					0.2528123 (0.3784524)	p<0.001
Cigarette × residual					−0.5719998 (0.3828804)	
Alcohol × residual					0.6083365 (0.498787)	p=0.03
Observations	25730		25730		25730	
Pseudo *R* ^2^	0.3674		0.3705		0.3402	

*Note*: Standard errors in brackets.

Abbreviations: CFA, control function approach; NCDs, noncommunicable diseases; 2SRI, two‐stage residual inclusion.

*Source*: Author's computation, 2023.

The analysis introduced the interactions to address the heterogeneity between the residuals and their respective endogenous variables within the model by incorporating additional variables while estimating the structural equation. Consequently, interaction terms were established for variables such as alcohol consumption and fruit/vegetable intake about their corresponding residuals. Conversely, the coefficient for cigarette smoking, along with its interaction term and residuals, was found to be insignificant. It suggests that heterogeneity may not be a significant concern when considering the interaction between cigarette smoking and its associated variables. However, significant interaction terms were observed for vegetable intake and alcohol consumption, indicating the presence of heterogeneity resulting from the interaction between the analyzed endogenous variables and unobserved risk factors for NCDs.

The CFA estimates reveal that the coefficient of income is positive, indicating a positive correlation between income and NCDs. It implies that households with higher incomes are at greater risk of developing NCDs compared to those with lower incomes, although the likelihood of NCD occurrence increases at a decreasing rate as income rises, implying that the risk of NCDs is lower among the wealthiest individuals.

Furthermore, the findings presented in Table 4 indicate that alcohol consumption and cigarette smoking have a positive and significant correlation with the NCDs indicating that increased consumption of alcohol and cigarette smoking causes the development of the NCDs. These findings are consistent with studies conducted by Ahmed et al.^29^ and Dalal et al.,^30^ which emphasize the strong link between NCDs in developing countries and behavioral risk factors. On the other hand, the consumption of fruits and vegetables is found to have a negative and significant correlation to the NCD, implying that sufficient intake or consumption of fruits and vegetables helps reduce the NCD development rate across households. Studies in other developing countries argued that increasing fruit and vegetable intake can reduce the likelihood of NCDs by as much as 71%.^31^, ^32^, ^33^ Similar findings were also observed by Mwai (2014) in Kenya, who suggested that low vegetable and fruit consumption contributes to a 41% increase in the likelihood of developing NCDs.

Furthermore, Table 4 presents the correlation between proxy variables, including average cigarette smoking, alcohol consumption, fruit and vegetable intake, and the prevalence of NCDs. A rise of 1% on average cigarette smoking and alcohol consumption is associated with a corresponding increase of 1.888% and 0.864% in the likelihood of NCD occurrence, respectively. These findings suggest that smoking and alcohol consumption are influenced by social factors, such as neighborhood and peer influences, which heighten the risk of NCDs within households. These results align with previous studies conducted by Suls and Green,^34^ Larsen et al.,^35^ and Caudill and Kong,^36^ which highlight the impact of social imitation on alcohol consumption and cigarette smoking behaviors. They support that positive social interactions are persuasive, encouraging healthier behaviors like consuming nutritious foods and a well‐balanced diet.

The study also establishes the relationship between demographic characteristics (sex, years of schooling, age, and area of residence) and NCD risk factors. Findings show that being in urban has a positive and significant correlation to the development of NCDs compared to their rural counterparts. These findings support the findings of Tawa et al.,^25^ that urban residences exhibit a higher degree of risk factors contributing to NCD development.

Furthermore, the study finds that ageing in Tanzania has a positive correlation to NCDs, implying that older populations are more prone to NCDs than the young population. These results differ from those of Barikdar et al.^37^ and Dalstra et al.,^38^ who found that youths are more likely to get NCDs due to their lifestyles, primarily on excessive alcoholic consumption and insufficient time for physical exercise. On the other hand, although sex was not significant, it was found to have a negative correlation to the NCDs, implying that being male reduces NCDs compared to female counterparts. However, there is no consensus on whether sex type can influence the early development of NCDs. Yet, studies by Tawa et al.,^25^ Lima et al.,^39^ and Taylor et al.^40^ found that heart diseases develop more quickly among females than males.

Additionally, limited access to health services and low health promotion contribute to the vulnerability of households to NCDs.^10^ These arguments correlate with the findings of this study which show that as healthcare facilities are distant from household residents, it increases the chances for households to be endangered by diseases. The reason is the failure to get immediate healthcare consultation for disease symptoms, leading to more critical conditions or severe illnesses.

## 4 CONCLUSION

The findings indicate noteworthy associations between NCDs and various socioeconomic characteristics. Hereditary factors were also found to significantly influence the presence of NCDs, underlining the role of genetic predisposition in shaping health outcomes across diverse socioeconomic backgrounds. Additionally, alcohol consumption and cigarette smoking displayed statistically significant positive relationships with NCDs, emphasizing the adverse impacts of these practices on health. On the other hand, consuming fruits and vegetables exhibited a significant negative correlation with NCDs, suggesting that incorporating these nutrient‐rich foods into one's diet may reduce NCD risk, particularly among individuals with varying socioeconomic profiles. Furthermore, the study revealed that engaging in physical exercises had a negatively significant coefficient, indicating its potential to mitigate the risk of NCDs, highlighting the importance of an active lifestyle in promoting better health outcomes. From these findings, to lower the prevalence of NCDs, the following are recommendations:

Improved public awareness campaigns: A thorough public health campaign strategy that aims to raise awareness of the adverse effects of alcohol consumption and smoking should be developed and implemented. This program should target both urban and rural populations, highlighting how important it is to change these behaviors to prevent NCDs' development. Parallel to this, the campaigns should disseminate information on the benefits of including fruits and vegetables in daily diets, ultimately fostering an improvement in general well‐being.

Promotion of health and education: It is suggested that concerted efforts be made to encourage balanced diets, focusing on encouraging people to eat more fruits and vegetables. These activities may include educational resources, dietary advice, and more regionally focused initiatives like creating community gardens and farmers' markets. The goal is to increase fresh produce's availability and affordability, especially in urban areas where risk factors for NCDs are frequently more pronounced.

Reinforcement of healthcare infrastructure: Investing significantly in healthcare facilities and improving access to medical care, especially in remote and underserved areas, is crucial. It will ensure early detection and successful treatment of NCDs and improve health outcomes because of improvement in accessibility. Therefore, this calls for the development of new telemedicine and mobile healthcare technologies and the expansion of primary healthcare services and training programs for healthcare professionals.

Enacting policy interventions: To reduce alcohol and tobacco use, it is essential to advocate forcefully for laws and regulations that are supported by research. It includes actions like raising taxes on alcohol and tobacco products, enforcing strict rules on advertising techniques, and launching all‐encompassing programs to help people quit smoking. In addition, it is necessary to develop policies that support sustainable food systems, ensuring that wholesome foods, including fruits and vegetables, are widely accessible and economically viable for all socioeconomic groups.

By implementing these suggested policy changes based on the above recommendations, a concerted effort can be made to address the risk factors linked to NCDs, setting the course for reducing their prevalence and easing the burden they place on society. In the ongoing struggle against the scourge of NCDs, these initiatives can have a profound and lasting impact when coordinated effectively at both the local and global levels in Tanzania.

## 5 LIMITATION OF THE STUDY

Even though this study offers insightful information about the connection between risk factors and NCDs, it is important to recognize some limitations. Despite efforts to address endogeneity and heterogeneity using appropriate regression techniques, there may still be unobserved variables or factors that affect the relationship between risk factors and NCDs. The validity of the estimated effects may be impacted by omitted variable bias. However, the study's cross‐sectional design makes it difficult to prove a connection between risk factors and NCDs. Experimental or longitudinal studies would offer more decisive proof of causality and the temporal sequence of the observed associations.

There is a risk of recall or social desirability bias when factors like alcohol consumption, smoking habits, and dietary intake depend on self‐reported data. Participants might under‐ or overreport these behaviors, making the results inaccurate. The study might not have considered all external influences or confounding variables that could affect the link between risk factors and NCDs. These unmeasured variables might introduce bias and impact how the results are interpreted.

An in‐depth comprehension of the study's scope and implications requires an awareness of these limitations. Future studies should address these issues and offer more details on the intricate connection between risk factors and NCDs.

## AUTHOR CONTRIBUTIONS

**Felician A. Kitole**: Conceptualization; data curation; formal analysis; methodology; software; validation; visualization; writing—original draft. **Jennifer K. Sesabo**: Data curation; methodology; supervision; validation; writing—review and editing. **Robert M. Lihawa**: Conceptualization; writing—review and editing.

## CONFLICT OF INTEREST STATEMENT

The authors declare no conflict of interest.

## TRANSPARENCY STATEMENT

The lead author Felician A. Kitole, affirms that this manuscript is an honest, accurate, and transparent account of the study being reported; that no important aspects of the study have been omitted; and that any discrepancies from the study as planned (and, if relevant, registered) have been explained.

## Data Availability

The final data and codes used in the study are available upon reasonable request from the corresponding author.

## APPENDIX 1 Wald exogeneity test

**Table:**

Variable	*χ* ^2^ statistics	*d* f	*p* Value	Conclusion
Income = 0	7.456	1	0.005	Reject $H_{0}$ (endogeneity)

## APPENDIX 2 First‐stage regression summary statistics

**Table:**

Variable	*R* ^2^	Adjusted *R* ^2^	Partial *R* ^2^	Prob > *F*
Distance to water source	0.6537	0.5621	0.5388	p<0.001

## APPENDIX 3 Instrument strength by eigenvalue statistic

**Table:**

	Critical values			
2SLS relative bias	5%	10%	20%	30%
	10.40	6.61	3.12	1.33
	10%	15%	20%	25%
2SLS size of nominal 5% Wald test	29.13	22.34	14.08	11.12
LIML size of nominal 5% Wald test	10.29	7.02	5.05	2.17

Eigenvalue statistic = 57.89.

$H_{0}:\mathrm{Instruments}\mathrm{are}\mathrm{weak}$.

## APPENDIX 4 Test of overidentifying restrictions

**Table:**

Tests	Test scores	*p* Value
Sargan (score) *χ* ^2^ (2)	35.0042	p>0.99
Basmann *χ* ^2^ (2)	28.6433	p>0.99

$H_{0}:\mathrm{Instruments}\mathrm{are}\mathrm{not}\mathrm{valid}$.
